# Increase of Plasma Biomarkers in Friedreich's Ataxia: Potential Insights into Disease Pathology

**DOI:** 10.1002/mds.30250

**Published:** 2025-06-11

**Authors:** Christian Rummey, Gilbert Thomas‐Black, Hector Garcia‐Moreno, David R. Lynch, Rosella Abeti, Huseyin Arisoy, Amanda Heslegrave, Henrik Zetterberg, Paola Giunti, the European Friedreich's Ataxia Consortium for Translational Studies (EFACTS), Jörg Bernhard Schulz, Kathrin Reetz, Claire Didszun, Thomas Klockgether, Ilaria Giordano, Massimo Pandolfo, Chantal Depondt, Myriam Rai, Sylvia Boesch, Wolfgang Nachbauer, Andreas Eigentler, Elisabetta Indelicato, Paola Giunti, Michael Parkinson, Katarina Manso, Gilbert Thomas‐Black, Hector Garcia‐Moreno, Nita Solanky, Rosella Abeti, James Polke, Robin Labrum, Francisco Rodríguez de Rivera Garrido, Javier Mascias, Sara Sánchez Velasco, Sergio Secades García, Caterina Mariotti, Lorenzo Nanetti, Anna Castaldo, Alessia Mongelli, Mario Fichera, Thomas Klopstock, Ivan Karin, Claudia Stendel, Florentine Radelfahr, Alexandra Durr, Perrine Charles, Claire Ewenczyk, Jennifer Just, Georgios Koutsis, Richard Walsh, Enrico Bertini

**Affiliations:** ^1^ Clinical Data Science GmbH Basel Switzerland; ^2^ The Ataxia Centre, Department of Clinical and Movement Neurosciences University College London London United Kingdom; ^3^ National Hospital for Neurology and Neurosurgery, University College London Hospitals Foundation NHS Trust London United Kingdom; ^4^ Department of Pediatrics and Neurology Children's Hospital of Philadelphia Philadelphia Pennsylvania USA; ^5^ Department of Neurodegenerative Disease UCL Institute of Neurology London United Kingdom; ^6^ Dementia Research Institute London United Kingdom; ^7^ Clinical Neurochemistry Laboratory, Sahlgrenska University Hospital Mölndal Sweden; ^8^ Department of Psychiatry and Neurochemistry Institute of Neuroscience and Physiology, The Sahlgrenska Academy at the University of Gothenburg Mölndal Sweden

**Keywords:** ataxia, biomarkers, Friedreich's ataxia, neurofilament light

## Abstract

**Background:**

Therapeutic interventions in Friedreich's ataxia (FRDA) are progressing into clinical trials, and the need for robust and easily accessible biomarkers has arisen.

**Objective:**

This study aimed to consolidate preliminary findings of changes in the levels of neurofilament light (NfL), glial fibrillary acidic protein (GFAP), Tau, and ubiquitin C‐terminal hydrolase L1(UCH‐L1) in FRDA comparing two large independent cohorts of patients with healthy controls.

**Methods:**

Plasma samples of patients from two large natural history studies (n = 187) and a similar sized cohort of healthy control subjects (n = 127) were assessed using single‐molecule array measurements. Age‐adjusted biomarker levels were related to patients’ genetic profile, clinical progression, and comorbidities, and compared with controls. Sensitivity and specificity were assessed using receiver operating characteristic analyses.

**Results:**

NfL levels were significantly elevated in patients with FRDA younger than 40 years, showing a distinct age‐dependent trajectory: levels declined with age in FRDA but increased in controls. Longitudinal data indicated annual NfL reductions between 8% (children) and 13% (young adults < 35 years). This result is discussed in the context of other neurodegenerative conditions, with FRDA being a rare case where NfL levels decrease over time before the age of 40 years. In contrast, tau was consistently elevated across all age groups in FRDA.

**Conclusions:**

NfL is a sensitive biomarker in early FRDA but decreases with age, converging with control values after 35–40 years. This age‐dependent pattern must be considered when interpreting the effect of interventions in clinical trials. Especially in younger (age < 10 years) or presymptomatic patients and control subjects, additional longitudinal sampling is warranted. Elevated tau levels suggest involvement in underlying disease pathophysiology. © 2025 The Author(s). *Movement Disorders* published by Wiley Periodicals LLC on behalf of International Parkinson and Movement Disorder Society.

Friedreich's ataxia (FRDA), an autosomal recessive disorder, is the commonest hereditary ataxia, accounting for half of all cases and three quarters of those younger than 25 years. The cause is an inherited unstable GAA expansion in intron 1 of the *FXN* gene.^1^ Frataxin is a mitochondrial protein involved in a variety of cellular processes, including Fe‐S cluster biosynthesis, iron homeostasis, and control of reactive oxygen species,^2^ with its deficiency resulting in mitochondrial energy imbalance, increased oxidative stress, and lipid peroxidation.^3^ Various neurological and nonneurological symptoms are seen in FRDA, chiefly a slowly progressive, mixed sensory and cerebellar ataxia. Other cardinal features include dysarthria, peripheral neuropathy, scoliosis, pes cavus and hypertrophic cardiomyopathy.^4^

There is a growing need to identify robust and accessible biomarkers to monitor disease activity and therapeutic efficacy in FRDA,^5^ especially given the recent advancements in this field.^6^, ^7^ Biomarkers are defined by the US Food and Drug Administration (FDA) as objectively measurable characteristics that are indicators of physiological and pathological processes or reflect response to therapeutic interventions.^8^ A recent large systematic review of therapeutic biomarkers across several domains (including biochemical, cardiac, clinical, and imaging outcomes) concluded that the Friedreich's Ataxia Rating Scale (FARS and modified FARS [mFARS]) and possibly left ventricular mass index were the only reliable biomarkers for monitoring therapeutic response, and notably did not identify any potential biofluid biomarkers.^9^ Notably, none of the studies included analyzed the biomarkers studied in this research project.

Biofluid biomarker research in FRDA has followed two avenues of exploration: the first concentrating on measuring frataxin and the second assaying downstream pathological markers of mitochondrial dysfunction and oxidative stress. In this study, we examine a third avenue: proteins released from neurons or glia that cross the blood–brain barrier into plasma.

Given that FRDA is a neurodegenerative disorder, investigating central nervous system (CNS)‐specific proteins reflecting damage in different CNS cell types appears appropriate. Over the past two decades, several proteins have emerged as promising biomarkers for neurodegeneration.^10^ These include: (1) neurofilament light (NfL),^11^ a neuron‐specific cytoskeletal protein^11^; (2) glial fibrillary acidic protein (GFAP), an astrocytic intermediate filament protein; (3) ubiquitin C‐terminal hydrolase L1 (UCH‐L1), an enzyme expressed in the cytoplasm of neurons^12^; and (4) tau, a microtubule‐associated protein.^13^ These proteins are released after neuronal or glial injury (and to a lesser extent as part of normal cellular turnover and growth or plasma membrane fragility) and subsequently detectable in the cerebrospinal fluid (CSF) and plasma. Methodologies such as the single‐molecule array (Simoa) technology have enabled reliable measurements of these proteins in plasma to the low picogram per milliliter level.

Our previous analysis showed increased plasma NfL, GFAP, and UCH‐L1, but not t‐tau, in patients with FRDA.^14^ No correlations with the number of repeats in the shorter allele (GAA1) nor the Scale for Assessment and Rating of Ataxia (SARA) score were found. In this study, we aimed to confirm these findings in a larger cohort including participants of different geographical origin and with a larger range of age at symptom onset (AAO), GAA1, comorbidities, disease duration, and functional status as measured by disease stage, as well as SARA and mFARS score.

## Subjects and Methods

### Samples

Plasma samples from 89 patients with FRDA enrolled in the European Friedreich's Ataxia Consortium for Translational Studies (EFACTS) (NCT02069509, REC No.: 10/H0716/51) natural history study were provided from the central sample repository. A second cohort of 98 FRDA plasma samples (of whom 64 had follow‐up samples) from patients participating in the Collaborative Clinical Research Network in Friedreich's Ataxia (CCRN in FA; NCT03090789) were also provided; these patients were also enrolled in the FACOMS natural history study. Samples covered the full spectrum of disease stages as defined by neurological scores (SARA or mFARS), age at onset, and shorter allele repeat length (GAA1). Repeat length in the longer allele (GAA2) correlates minimally with disease stage or progression.^15^ Further demographic and clinical data were obtained from the EFACTS and Friedreich's Ataxia Clincial Outcome Measures (FACOMS) registries/natural history studies.

A total of 127 plasma samples from control subjects (no neurological condition) across a wide age range were provided by University College London (UCL) Hospitals (UCLH) Bioresource and the Arthritis Research UK Centre for Adolescent Rheumatology at UCL, UCLH.

### Measurement of Plasma Biomarkers

Plasma NfL, GFAP, tau (hereafter referred to as total tau [t‐tau]), and UCH‐L1 concentrations were measured using the Simoa Human Neurology A 4‐Plex assay (N4PA) (Quanterix, Billerica, MA, USA) on the Simoa HD‐1 Analyser (Quanterix, Billerica, MA, USA) according to the manufacturer's guidelines. The lower limits of detection for the proteins are 0.104, 0.221, 0.024, and 1.74 pg/mL for NfL, GFAP, t‐tau, and UCH‐L1, respectively. Samples were measured in duplicate by aspirating twice from a single well. The mean of the two measurements was used for analysis. Mean coefficients of variation (CVs) were acceptable for NfL (4.7%), GFAP (3.1%), and t‐tau (9.5%). However, for UCH‐L1, the mean CV was 37%, with 183 samples (48%) missing the criterion of 20%. UCH‐L1 results are therefore reported with caution. Other investigations using this assay have found similar results.^16^

### Statistical Analysis

#### Biomarker Levels

All biomarker levels were log transformed because of nonnormal distribution, as previously reported.^14^, ^17^ Progression with age was evaluated using graphical methods and linear mixed effect models (using repeated measures), with age as linear or polynomial effect. Models were selected based on visual inspection and fit statistics. Differences between the FRDA and control groups were evaluated using fixed effects contrasts and interaction effects.

Sensitivity and specificity to separate patients with FRDA from control subjects were evaluated using receiver operating characteristic (ROC) curves at different age cutoffs.

In patients with FRDA, we tested the impact of AAO or GAA1 on biomarker values because both these measures affect progression. Individual biomarker values were tested for their impact on our results with two separate methods (*z* scores and cook's D), but in none of our analyses could a relevant impact on the model results be found. Therefore, although some extreme values could not be explained, no outliers were excluded from the analyses. In addition, NfL levels were correlated with neurological examination scores (SARA and mFARS) and ambulatory status. Furthermore, typical comorbidities in FRDA that were recorded both in EFACTS and in FACOMS (diabetes, optic atrophy, and cardiomyopathy) were evaluated for their influence on NfL levels in separate models using comorbidities (0 or 1) as fixed factors in the NfL models.

All *P* values are provided for orientation only and without a specific significance level or adjustment for multiple testing. All statistical calculations were performed in R (R Core Team, 2019) using tidyverse and lme4 for mixed effect models.

### Standard Protocol Approval, Registration, and Patient Consent

This study has been approved by respective institutional review boards, and all subjects enrolled provided informed consent before participation.

## Results

### Demographic Characteristics

To ensure a broad coverage of disease characteristics, we combined samples from EFACTS^18^ and FACOMS^15^ cohorts (Table 1). The former primarily recruited participants older than 18 years, whereas the latter focused more on children. As a result, EFACTS patients were older (*P* < 0.001) with a trend to longer disease duration (*P* = 0.106). Overall, participants predominantly had an early onset (age of onset, <15 years; n = 112, 59.9%), with slightly more late‐onset participants from the EFACTS cohort. Thus, the two subgroups were complementary. Participant's overall median age was 31 years (interquartile range [IQR], 21–42 years; range, 8–84 years) with a median disease duration of 15 years (IQR, 8–23 years; range, 1–55 years).

**TABLE 1:** Demographic characteristics of participants with FRDA, by study and overall

	FACOMS	EFACTS	Overall	Controls
N	98	89	187	127
Female sex, n (%)	52 (53.1)	47 (52.8)	99 (52.9)	67 (52.7)
Age, y [IQR]	26 [19, 34]	37 [27, 44]	31 [21, 42]	
Disease duration, y [IQR]	14 [8, 21]	19 [9, 27]	15 [8, 23]	
Age of symptom onset, y [IQR]	12 [8, 16]	14 [11, 23]	13 [10, 18]	
GAA1 [IQR]	620 [470, 789]	585 [367, 767]	600 [400, 768]	
Compound heterozygous, n (%)	1 (1.0)		1 (0.5)	
Age at symptom (grouped), n (%)				
Early (<15 y) n(%)	63 (64.3)	49 (55.1)	112 (59.9)	
Intermediate (15–24 y) n(%)	25 (25.5)	20 (22.5)	45 (24.1)	
Late (>24 y) n(%)	10 (10.2)	20 (22.5)	30 (16.0)	

Abbreviations: FRDA, Friedreich's ataxia.

We were interested in consistent biomarker slopes over the complete age range, and control subjects were selected to represent a balanced distribution over age (median, 51 years; IQR, 35–64 years; range, 13–84 years). For NfL, linear slopes with age have been reported.^17^

### Biomarker Levels in Control Subjects Versus Patients with FRDA

**FIG. 1:**
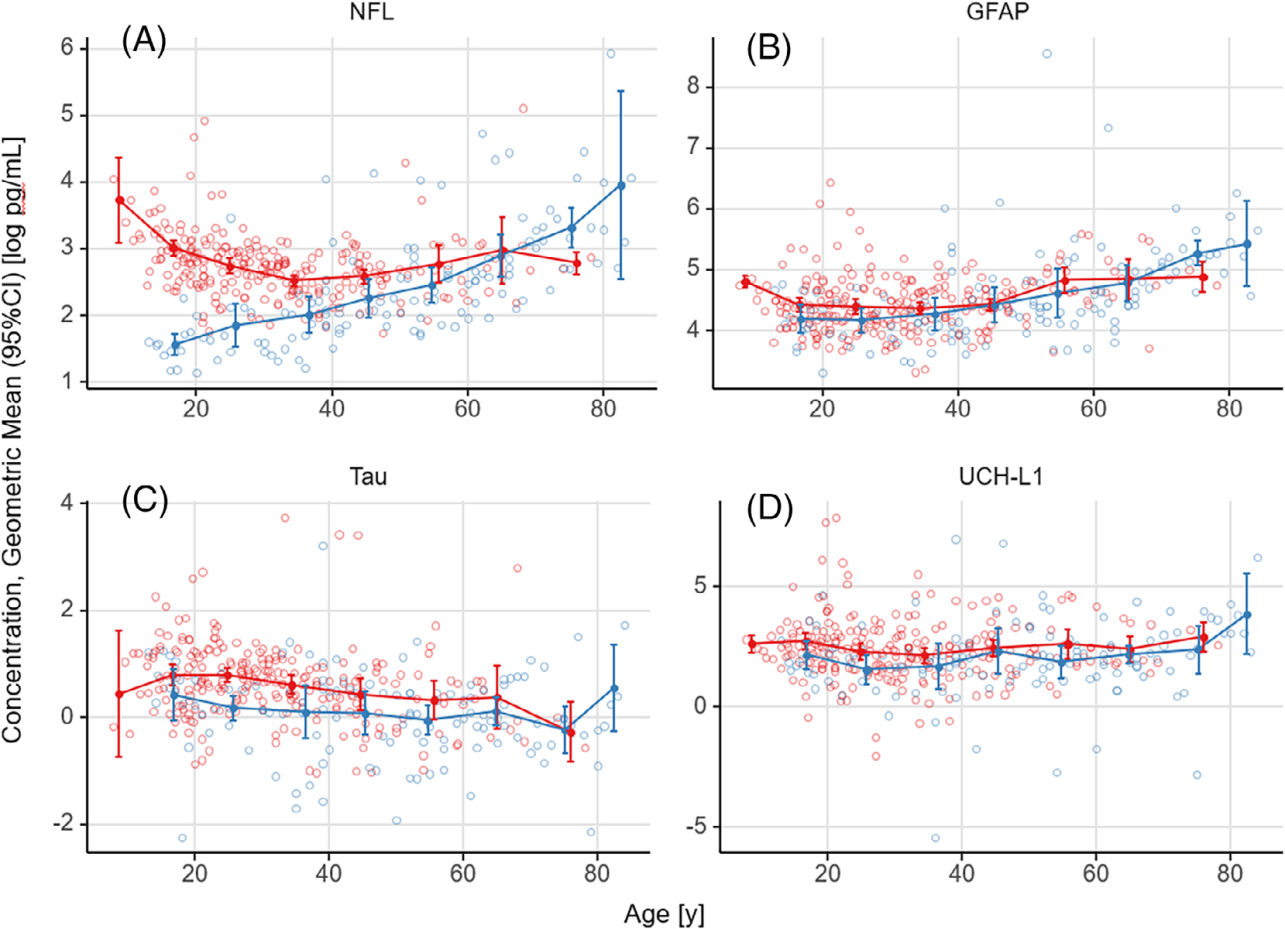
Biomarker levels in participants with Friedreich's ataxia (FRDA) (red) and control subjects (blue) by age. Mean lines for samples within 5‐year age bins are depicted in connected dots with 95% confidence intervals (CIs); individual measures are shown as circles: (**A**) neurofilament light [NfL], (**B**) glial fibrillary acidic protein [GFAP], (**C**) t‐tau, and (**D**) ubiquitin C‐terminal hydrolase L1 (UCH‐L1). [Color figure can be viewed at wileyonlinelibrary.com]

Scatterplots by age of all biomarker samples in patients with FRDA versus control subjects are shown in Figure 1 and Fig. S1, together with mean levels calculated within 5‐year age brackets to indicate progression over age. Linear mixed effects modeling results using biomarker levels as dependent and age as independent variable are summarized in Table 1.

Our control group (n = 127) confirmed previous findings for all biomarkers in healthy subjects. NfL increased linearly with age by 0.031 log pg/mL per year (95% confidence interval [CI]: 0.025, 0.036; *P* < 0.001). GFAP levels also increased slightly with age, by 0.018 log pg/mL per year (95% CI: 0.012, 0.024; *P* < 0.001), whereas t‐tau (0.006 log pg/mL per year; 95% CI: −0.013, 0.002; *P* = 0.127) and UCH‐L1 levels (0.013 log pg/mL per year; 95% CI: −0.002, 0.028; *P* = 0.087) in control subjects did not change significantly over the investigated age range (Table S1).

Age‐adjusted differences between patients with FRDA and control subjects were found for NfL (−0.497 log pg/mL; 95% CI: −0.639, −0.355; *P* < 0.001) and t‐tau (−0.384 log pg/mL; 95% CI: −0.561, −0.206; *P* < 0.001), whereas the overall differences in GFAP and UCH‐L1 were small (−0.096 log pg/mL; 95% CI: −0.229, 0.038; *P* = 0.163; and −0.359 log pg/ml; 95% CI: −0.706, −0.011; *P* = 0.044, respectively). For younger patients (<13 years old), however, GFAP levels seemed increased for FRDA (Table S1). Unfortunately, no age‐matched controls were available for that range of ages (see later and Fig. 1).

From visual inspection, GFAP, t‐tau, and UCH‐L1 levels showed a linear change with age in both groups (Fig. 1). NfL in control subjects also followed a linear pattern, supporting the model in Table 1. In patients with FRDA, however, NfL followed a second‐order polynomial slope. Thus, GAA1 repeat length and age‐of‐onset effects on NfL were evaluated using a second‐order polynomial model.

### Analysis of Follow‐up Samples

**FIG. 2:**
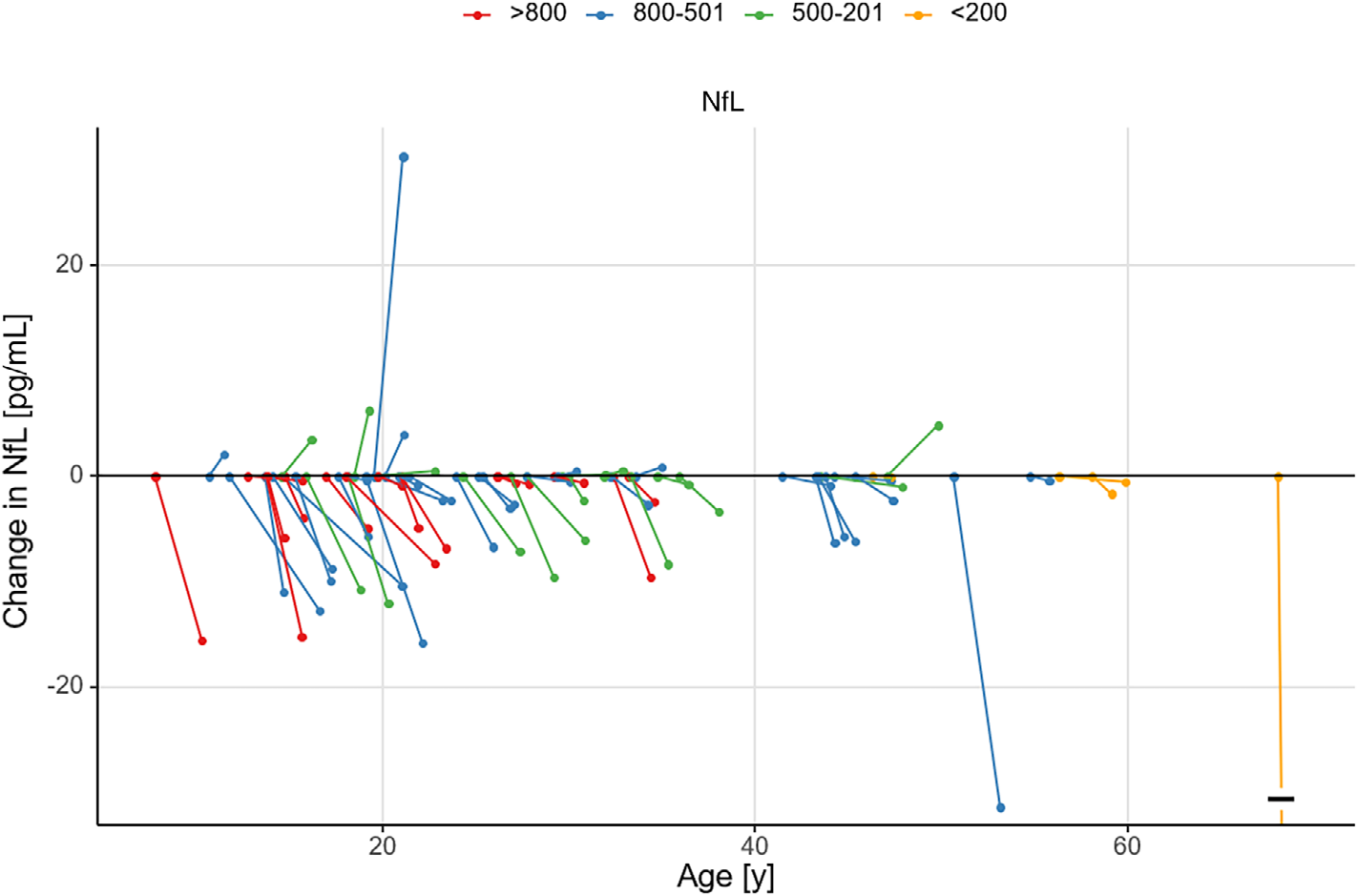
Changes in neurofilament light (NfL) levels respective to age, color coded by number of GAA1 repeat length. The *y*‐axis was limited to ±30 pg/mL (one sample changed by −150 pg/mL at age 69 years). [Color figure can be viewed at wileyonlinelibrary.com]

In 64 patients with follow‐up samples, the mean interval was 2.1 years (range, 0.8–6.4 years). From the mixed model results, GFAP, t‐tau, and UCH‐L1 change only minimally over age (Table 1). When categorizing patients into two groups, that is, increasing versus decreasing levels, for GFAP, 50% of patients had decreasing levels and 50% had increasing levels. Respective numbers for t‐tau were 58% versus 42% and for UCH‐L1 were 54% versus 46%. These distributions were age independent (data not shown). NfL levels, in contrast, declined in 83% and increased in only 17% of patients with follow‐up samples (Fig. 2). Two patients changed by an absolute number greater than 20 pg/mL, and one patient showed a change of 150 pg/mL. *After excluding outliers, the annualized geometric mean ratio was 0.87 (13% decrease per year) i*n the pediatric group (n = 15; annualized mean change = −3.5 pg/mL per year) and 0.93 (7% decrease per year) in the young adults aged 18–35 years (n = 33; annualized mean change = −1.3 pg/mL per year). In patients older than 35 years, NfL levels did not discriminate between patients with FRDA and control subjects (Fig. 1), although patients with FRDA aged older than 35 years declined by 10% on average (n = 13; annualized geometric mean ratio, 0.90; annualized mean change, −1.5 pg/mL) (Table S2).

### Sensitivity and Specificity

**FIG. 3:**
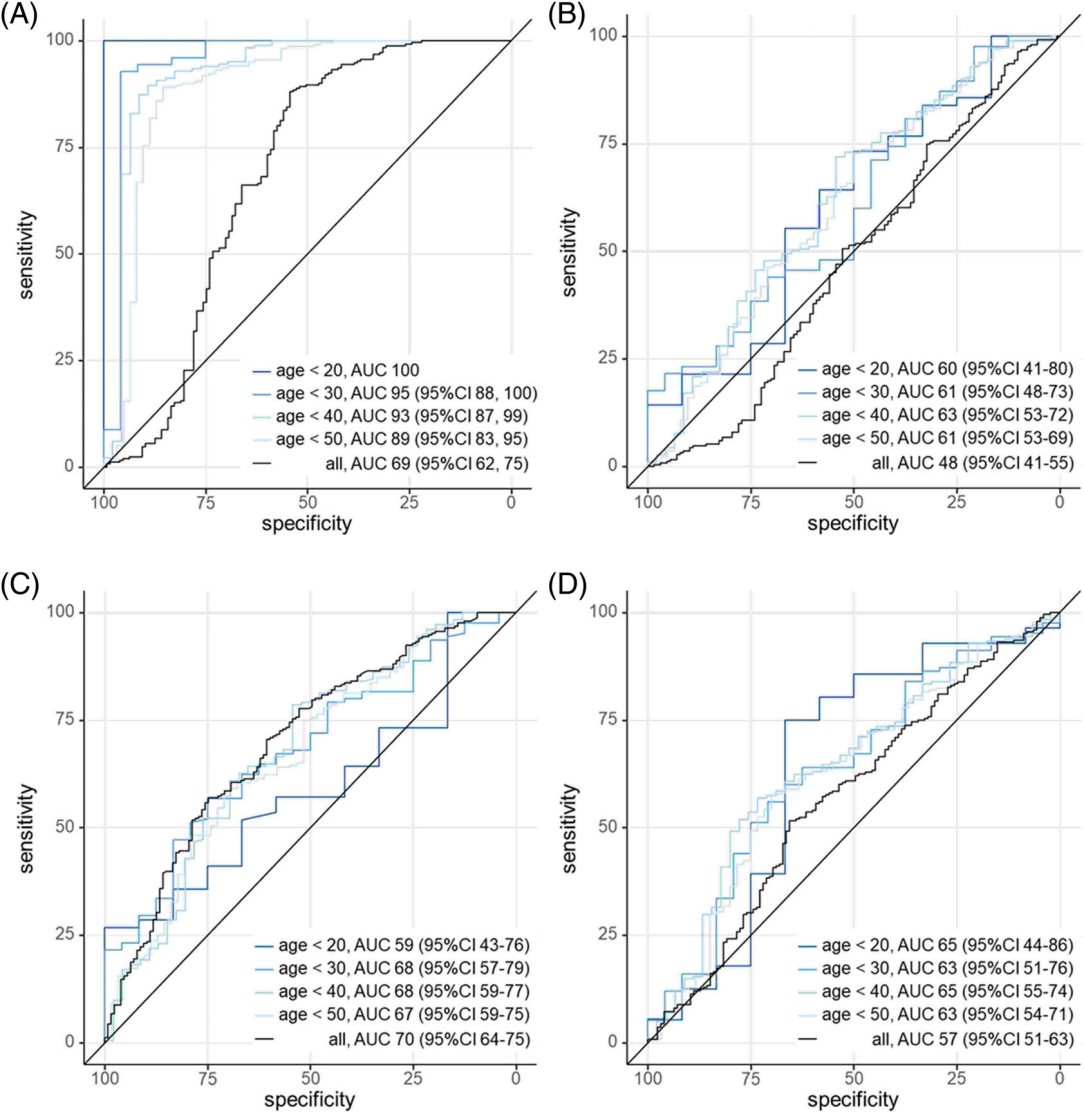
Receiver operating characteristic (ROC) curves for neurofilament light (NfL) (**A**), glial fibrillary acidic protein (GFAP) (**B**), t‐tau (**C**), and ubiquitin C‐terminal hydrolase L1 [UCH‐L1] (**D**); different age cutoffs are depicted by color, with resulting area under the curve (AUC) (%) results. CI, confidence interval. [Color figure can be viewed at wileyonlinelibrary.com]

To investigate the capability of the four biomarkers to separate FRDA and control samples, we calculated ROC curves for separate age cutoffs (Fig. 3). In subjects younger than 20 years, NfL level perfectly separated controls from FRDA (area under the curve [AUC] = 100%), declining to 89% (95% CI: 83%–95%) at ages <50 years and 69% (95% CI: 62%–75%) for all ages. Of the other three biomarkers, only t‐tau showed a modest AUC (70% over all ages), whereas GFAP and UCH‐L1 did not prove to be specific at any ages (Fig. 3).

### NfL Levels Are Affected by GAA1

As described earlier, progression of NfL levels in patients with FRDA can be described by a second‐order polynomial curve. From the respective random coefficient regression model over the complete age range, FRDA NfL levels were higher than in controls (2.56 vs. 2.01 log pg/mL; *P* < 0.001), with limited specificity for FRDA samples at ages older than 40 years.

**FIG. 4:**
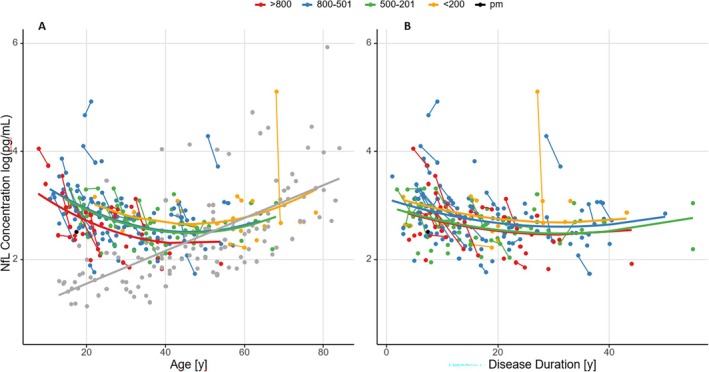
Neurofilament light (NfL) levels in control subjects (gray) and patients with Friedreich's ataxia (FRDA) relative to age (left) and disease duration (right). Patients with FRDA were grouped and colored by GAA1 repeat length. Solid lines depict polynomial model functions of NfL levels, by respective GAA1‐repeat length group. Control levels were modeled using a linear function over age. [Color figure can be viewed at wileyonlinelibrary.com]

Similar second‐order polynomial models with NfL as dependent variable were used to assess the impact of AAO and GAA1. Age of onset had no impact on NfL levels (*P* = 0.799), whereas GAA1 predicted NfL (*P* = 0.0134). Notably, the regression coefficient for GAA1 was *negative* (−0.035 log [NfL (pg/mL)] per 100 repeats), indicating lower NfL levels for longer GAA1 repeat lengths, as shown in Figure 4A and Fig. S2A.

A similar model using disease duration as time variable showed that age is the predominant factor in the decline of NfL, and that the levels do not depend on disease duration. Also, the relationship between NfL levels and GAA1 repeat length was less pronounced in this model (Fig. 4B and Fig. S2B).

### Neurological Status and FRDA‐Related Comorbidities

Correlations of NfL levels with neurological scores, as well as by ambulatory status or FRDA‐related comorbidities, were investigated: 20 patients (10.6%) had diabetes, 12 patients (6.5%) had optic atrophy, and a total of 106 (57%) patients had cardiomyopathy. No correlations with biomarker levels were found (data not shown).

## Discussion

Our study investigated plasma levels of four CNS‐specific biomarkers in patients with FRDA to confirm results from a pilot experiment^14^ in a larger, diverse sample using age‐adjusted analyses. Follow‐up samples from FRDA participants were compared with control samples from a large cohort of healthy control subjects (n = 127) using linear mixed effect models. Sensitivity and specificity to differentiate FRDA samples from controls were evaluated using ROC analyses stratified by age. Finally, our large control cohort documents the age dependency of these biomarkers.

Our results indicated increased levels in patients versus control subjects for NfL and t‐tau. In NfL, these differences were pronounced and potentially useful in young patients but diminished around an age of 40 years. In contrast, for t‐tau, the difference was smaller yet consistent across all ages. For UCH‐L1, no statistically significant differences versus controls were found, but our UCH‐L1 results lacked convincing reproducibility.

Although based on a small sample, GFAP levels appeared elevated in very young patients (<13 years),^14^ although age‐matched control data were unavailable. One study on brain injury biomarkers in pediatric trauma patients without head injury or concussion (median age, 9 ± 4 years) found serum GFAP levels <0.1 ng/mL,^19^ a result also confirmed in slightly older children with orthopedic injuries.^20^ It is therefore conceivable that elevated GFAP levels occur in FRDA children younger than 13 years.

Tau levels in FRDA were moderately elevated compared with controls. Tau is released during axonal degeneration and turnover,^21^ and increased turnover may reflect CNS attempts to compensate for degeneration by forming new circuits.^22^, ^23^ This may also occur in the peripheral nervous system (PNS), as seen in rats with sciatic nerve crush injury, where tau mRNA and protein initially decreased but later significantly increased.^24^ Furthermore, tau protein isoforms differ between plasma and CSF, with full‐length tau being dominant in plasma, but not in the CSF. This suggests peripheral sources for the majority of plasma tau, rather than CSF^25^ (our assay does not discriminate between tau isoforms). Interestingly, tau levels decreased with age in patients with FRDA but remained unchanged in controls, possibly reflecting exhaustion of a regenerative compensatory mechanism in the CNS and PNS.

We found clear differences in the levels of NfL between FRDA and controls, confirming the result from our previous study,^14^ a different EFACTS cohort,^26^ and a study conducted at the Children's Hospital of Philadelphia.^27^

Under normal conditions, neurofilaments are stable within axons and essential for radial growth and axonal stability (their precise function remains unclear^28^, ^29^). Mildly increased NfL levels in healthy children stabilize by age 10 years and are thought to reflect a high cell turnover due to neuronal migration and differentiation.^30^ Younger than that age, elevated NfL levels could reflect neurodevelopmental processes, particularly in early‐onset FRDA, with levels remaining elevated until age 35–40 years. Such a change in the pathophysiology after a certain age is supported by another recent study. In FRDA, after 35 years of age, the telomere length is also longer compared with controls, whereas the opposite is true thereafter, the significance of which needs to be further explored.^31^

In patients older than 35–40 years, NfL levels do not discriminate FRDA samples from controls, and known predictors of clinical status and progression showed minor (GAA1) or no impact (AAO) on NfL levels. Our statistical modeling suggests age instead as the dominant driver of NfL levels, which was supported by follow‐up samples showing a general decline, especially in younger samples. This inverse correlation between age and NfL has been previously observed by Clay et al.^27^ Although sampling has been at irregular intervals, follow‐up analysis has shown a 13% decrease per year in the pediatric population (<18 years) and an 8% decrease in the young adult population (18–35 years). These values can serve as reference thresholds for expected natural changes in NfL, and deviations—stabilization or accelerated decline—may indicate biological treatment effects in clinical trials and need to be interpreted in the context of clinical outcomes. Notably, although the number of our follow‐up examples was low, even in patients aged >35 years, NfL levels continued to decline, unlike in controls where NfL levels increase with age.

The finding that longer GAA1 repeat lengths lead to lower levels of NfL is surprising but is supported by prior studies.^26^, ^27^ This could be because of degeneration or growth failure of affected nervous tissue resulting in increased NfL earlier in the disease, before diagnosis. In contrast, in polyQ diseases, the opposite has been reported, especially in preataxic carrier and ataxic stages of spinocerebellar ataxia type (SCA) 3,^32^, ^33^ as well as SCA1 and SCA7.^34^ Another explanation may be hypoplasia of affected tissues, as proposed by Koeppen et al,^35^ suggesting reduced tissue quantity available for degeneration and subsequent NfL release. These authors did not find an influence of GAA1 on thoracic spinal cord or nerve cell area of the dorsal root ganglion, likely because of the inclusion of subjects at the end stage of the disease (Disease duration = 28.1 ± 13.8). Our observation aligns with Santoro et al,^36^ who reported that the number of nerve fibers present in sural nerve biopsies was inversely correlated with GAA1, and that GAA1 was a significant determinant of large fiber loss. If peripheral nerve degeneration is the main driver of serum NfL levels, this relationship could explain our findings. We also did not find any correlation between comorbidities and the explored biomarkers.

The behavior of NfL levels over time can vary between neurodegenerative diseases,^17^, ^37^, ^38^, ^39^ but usually they increase or remain constant over time and with disease manifestation, as shown in, for example, genetic frontotemporal dementia,^39^, ^40^ sporadic Creutzfeldt‐Jakob disease,^41^ Charcot‐Marie‐Tooth disease,^42^ Huntington's disease,^17^ familial amyotrophic lateral sclerosis,^43^ and autosomal dominant Alzheimer's disease,^44^ as well as other genetic ataxias such as RFC1 disease^45^ and certain spinocerebellar ataxias.^32^, ^33^, ^34^ This is also true for Multi System Atrophy (MSA), in which NfL levels increase in the first 7 years of disease duration and gradually reduce thereafter.^46^, ^47^ FRDA appears to be the only neurodegenerative condition where NfL levels decrease with increasing age below the age of 40 years without treatment. Hayer et al^26^ found stable NfL levels over 2 years. The higher median age and less representation of younger samples in their study could explain the discrepancy. A comprehensive table summarising other behavior of the Nfl in differnt neurodegeneartive diseaseses is reported in suplemantary data. (See Supporting information Data S1)

There are several (nonexclusive) possible explanations for the phenomenon of declining NfL levels. A recent mitochondrial biomarkers study showed that the increase in NfL levels in FRDA is unrelated to activity of electron transfer chain complexes, supporting an explanation independent of mitochondrial dysfunction.^48^ The rate of production of NfL declines over time. The extremely high levels in most young subjects could represent attempted regeneration by the nervous system, which subsequently declines as discussed earlier. The second possible explanation is an increased clearance rate. After an acute insult such as infarct or trauma, NfL levels increase over the course of 7 days^49^, ^50^ and return to baseline between 6 and 15 months after insult.^51^, ^52^ These studies suggest that either NfL is cleared relatively slowly after an acute insult, which makes an increased rate of clearance less likely as an explanation, or there is a period of neurogenesis and/or increased plasticity after an insult leading to the slow decline of NfL levels. It is important to note that in both mentioned, there may have been ongoing neurological injury that could have contributed to the slow decline of NfL and hence low imputed clearance rate. Because data regarding NfL's kinetics are scant, more detailed longitudinal studies are required to elucidate the true dynamics of NfL.

Another explanation is “burnout,” where the amount of neural tissue producing NfL declines steadily (assuming constant production and clearance rates) leading to a net decrease over time. This ongoing loss of neural tissues would be in keeping with the gradual worsening seen clinically. This concept of “burnout” is in keeping with findings from other neurodegenerative diseases, supporting the notion that NfL is a marker of current neuronal decay rather than being a function of disease progression.

NfL may also reflect underlying neuroinflammation, and not just neurodegeneration. A study measuring NfL levels in patients with autoimmune neurological disorders such as autoimmune encephalitis indicated raised levels in the acute phase that near normalized with immunotherapy.^53^ A predominantly neuroinflammation‐mediated increase in NfL would explain the trend observed in this study, supported by a recent imaging study showing a greater central neuroinflammatory load earlier in disease course^54^ and the increase of GFAP in the younger Friedreich's especially before 15 years of age at an early stage of the disease.

To further explore this, NfL levels in the various FRDA mouse models could prove revealing. Such data would enable the determination of NfL in presymptomatic disease stages and investigation of whether the changes seen in patients are recapitulated in transgenic mice. Interestingly, recent murine models of SCA3 show that NfL increase likely precedes onset of Purkinje cell loss.^32^

Besides the large control group, a clear limitation is the absence of very young controls; such samples are hard to obtain, yet many pediatric neurological conditions could benefit from normative biomarker data. Although NfL levels clearly diverge from controls, we cannot confirm increased GFAP in our very young patients because of this limitation. We are also unable to comment on the origins of NfL or tau in our cohort's plasma. NfL may be released from CNS axons into the CSF and from there into the blood, or from PNS axons.

To conclude, we confirm the finding of increased levels of NfL in young patients with FRDA and provide statistical models to guide quantification. This could be a first step toward the use of NfL in clinical trials, in combination with functional outcomes, similar to other diseases (like multiple sclerosis^55^, ^56^ and spinal muscular atrophy^57^, ^58^, ^59^). Nevertheless, a deeper understanding of the mechanism of elevation in FRDA (as discussed in a recent review^60^) is certainly required.

## Author Roles

Christian Rummey: Drafting/revision of the manuscript for content, including medical writing for content; study concept or design; and analysis or interpretation of data. Gilbert Thomas‐Black: Drafting/revision of the manuscript for content, including medical writing for content; major role in the acquisition of data; study concept or design; and analysis or interpretation of data. Hector Garcia‐Moreno: Drafting/revision of the manuscript for content, including medical writing for content; and major role in the acquisition of data. David R. Lynch: drafting/revision of the manuscript for content, including medical writing for content; major role in the acquisition of data; and analysis or interpretation of data. Rosella Abeti: drafting/revision of the manuscript for content, including medical writing for content. Huseyin Arisoy: Drafting/revision of the manuscript for content, including medical writing for content. Amanda Heslegrave: Drafting/revision of the manuscript for content, including medical writing for content. Henrik Zetterberg: Drafting/revision of the manuscript for content, including medical writing for content. Paola Giunti: Drafting/revision of the manuscript for content, including medical writing for content; major role in the acquisition of data; study concept or design; and major role in analysis or interpretation of data.

## Financial Disclosures for the Previous 12 Months

P.G. received funding from the European Commission Framework Project 7 (HEALTH‐F2‐2010‐242193). P.G. is also supported by the National Institute for Health Research University College London Hospitals Biomedical Research Centre UCLH. G.T.‐B. and P.G. work at UCLH/UCL, which receives a proportion of funding from the Department of Health's National Institute for Health Research Biomedical Research Centers funding scheme and receives support from the North Thames CRN. P.G. recieved funding from MRC grant code MR /N028767/1. The Simoa instrument was acquired through a Welcome Trust Multi‐User Equipment grant (to A.H. and H.Z.). H.Z. is a Wallenberg Scholar supported by grants from the Swedish Research Council (2018‐02532), the European Research Council (681712), Swedish State Support for Clinical Research (ALFGBG‐720931), and the UK Dementia Research Institute at UCL.

## Supporting information


**FIGURE S1.** Absolute biomarker levels in participants with FRDA (red) and controls (blue) by age. Mean lines for samples within 5 years‐age bins are depicted in connected dots with 95% CIs; individual measures are shown as circles (A: NfL, B: FAP, C: t‐tau and D: UCH‐L1).

**FIGURE S2.** Absolute NfL Levels in controls (grey) and FRDA patients relative to age (A) and disease duration (B). FRDA patients were grouped and coloured by GAA1 repeat length. Solid lines depict polynomial model functions of NfL levels, by respective GAA1‐repeat length group. Control levels were modelled using a linear function over age.

**Table S1.** Yearly Rates of change and age adjusted differences in NfL, GFAP, t‐tau and UCH‐L1 using linear mixed effects modelling.

**Table S2.** Annualized change in geometric mean ratio of NfL using follow‐up samples, analyzed by pediatric, young adult, and older adult age groups.

**Data S1.** Supporting Information.

## Data Availability

Anonymized data will be shared upon reasonable request of qualified investigators.

## Appendix A

Coinvestigators of the European Friedreich's Ataxia Consortium for Translational Studies (EFACTS)

Jörg Bernhard Schulz: Department of Neurology, RWTH Aachen University; JARA‐BRAIN; Institute Molecular Neuroscience and Neuroimaging, Forschungszentrum. Jülich GmbH and RWTH Aachen University, Germany (principal investigator, program coordinator). Kathrin Reetz: Department of Neurology, RWTH Aachen University; JARA‐BRAIN; Institute Molecular Neuroscience and Neuroimaging, Forschungszentrum. Jülich GmbH and RWTH Aachen University, Germany. Kathrin Fedosov: Department of Neurology, RWTH Aachen University, Germany (database oversight). Claire Didszun: Department of Neurology, RWTH Aachen University, Germany (database oversight). Thomas Klockgether: Department of Neurology, University Hospital of Bonn; German Center for Neurodegenerative Diseases (DZNE), Bonn, Germany (principal investigator). Ilaria Giordano: Department of Neurology, University Hospital of Bonn; German Center for Neurodegenerative Diseases (DZNE), Bonn, Germany (site investigator). Massimo Pandolfo: Laboratory of Experimental Neurology, Université Libre de Bruxelles, Brussels, Belgium (principal investigator). Chantal Depondt: Laboratory of Experimental Neurology, Université Libre de Bruxelles, Brussels, Belgium (site investigator). Myriam Rai: Laboratory of Experimental Neurology, Université Libre de Bruxelles, Brussels, Belgium (Genetics). Sylvia Boesch: Department of Neurology, Medical University Innsbruck, Austria (principal investigator). Wolfgang Nachbauer: Department of Neurology, Medical University Innsbruck, Austria (site investigator). Andreas Eigentler: Department of Neurology, Medical University Innsbruck, Austria (site investigator). Elisabetta Indelicato: Department of Neurology, Medical University Innsbruck, Austria (site investigator). Paola Giunti: Department of Molecular Neuroscience, Institute of Neurology, University College London, London, UK (principal investigator). Michael Parkinson: Department of Molecular Neuroscience, Institute of Neurology, University College London, London, UK (site investigator). Katarina Manso: Ataxia Centre, Department of Movement and Clinical Neurosciences, Institute of Neurology, University College London, London, UK (research nurse). Gilbert Thomas‐Black: Ataxia Centre, Department of Movement and Clinical Neurosciences, Institute of Neurology, University College London, London, UK (site investigator). Hector Garcia‐Moreno: Ataxia Centre, Department of Movement and Clinical Neurosciences, Institute of Neurology, University College London, London, UK (site investigator). Nita Solanky: Ataxia Centre, Department of Movement and Clinical Neurosciences, Institute of Neurology, University College London, London, UK (site investigator). Rosella Abeti: Ataxia Centre, Department of Movement and Clinical Neurosciences, Institute of Neurology, University College London, London, UK (site investigator). James Polke: Laboratory of Neurogenetic, The National Hospital for Neurology and Neurosurgery, NHNN/UCLH, Queen Square, WC1N 3BG London, UK (site investigator). Robin Labrum: Laboratory of Neurogenetic, The National Hospital for Neurology and Neurosurgery, NHNN/UCLH, Queen Square, WC1N 3BG London, UK (site investigator). Francisco Rodríguez de Rivera Garrido: Reference Unit of Hereditary Ataxias and Paraplegias, Department of Neurology, IdiPAZ, Hospital Universitario, La Paz, Madrid, Spain (principal investigator). Javier Mascias: Reference Unit of Hereditary Ataxias and Paraplegias, Department of Neurology, IdiPAZ, Hospital Universitario La Paz, Madrid, Spain (site investigator). Sara Sánchez Velasco: Reference Unit of Hereditary Ataxias and Paraplegias, Department of Neurology, IdiPAZ, Hospital Universitario, La Paz, Madrid, Spain (site investigator). Sergio Secades García: Reference Unit of Hereditary Ataxias and Paraplegias, Department of Neurology, IdiPAZ, Hospital Universitario La Paz, Madrid, Spain (site investigator). Caterina Mariotti: Unit of Medical Genetics and Neurogenetics, Fondazione IRCCS Istituto Neurologico, Carlo Besta, Milan, Italy (principal investigator). Lorenzo Nanetti: Unit of Medical Genetics and Neurogenetics, Fondazione IRCCS Istituto Neurologico, Carlo Besta, Milan, Italy (site investigator). Anna Castaldo: Unit of Medical Genetics and Neurogenetics, Fondazione IRCCS Istituto Neurologico, Carlo Besta, Milan, Italy (site investigator). Alessia Mongelli: Unit of Medical Genetics and Neurogenetics, Fondazione IRCCS Istituto Neurologico, Carlo Besta, Milan, Italy (site investigator). Mario Fichera: Unit of Medical Genetics and Neurogenetics, Fondazione IRCCS Istituto Neurologico, Carlo Besta, Milan, Italy (site investigator). Thomas Klopstock: Department of Neurology, Friedrich. Baur Institute, University Hospital of the Ludwig‐Maximilians‐Universität München; German Center for Neurodegenerative Diseases (DZNE), Munich; Munich Cluster for Systems Neurology (SyNergy), Munich, Germany (principal investigator). Ivan Karin: Department of Neurology, Friedrich Baur Institute, University Hospital of the Ludwig‐Maximilians‐Universität, München, Munich, Germany (site investigator). Claudia Stendel: Department of Neurology, Friedrich Baur Institute, University Hospital of the Ludwig‐Maximilians‐Universität, München, Munich, Germany (site investigator). Florentine Radelfahr: Department of Neurology, Friedrich Baur Institute, University Hospital of the Ludwig‐Maximilians‐Universität, München, Munich, Germany (site investigator). Alexandra Durr: Institut du Cerveau et de la Moelle épinière Sorbonne Université, INSERM U 1127, CNRS UMR 7225 and APHP, Pitié‐Salpêtrière, University Hospital, Genetic Department, Paris, France. Marie Biet: Institut du Cerveau et de la Moelle épinière, Sorbonne Université, INSERM U 1127, CNRS UMR 7225 (study coordinator). Perrine Charles: Pitié‐Salpêtrière. University Hospital, Paris, France (site investigator). Claire Ewenczyk: Pitié‐Salpêtrière University Hospital, Paris, France (site investigator). Jennifer Just: Department of Neurodegenerative Diseases, Hertie‐Institute for Clinical Brain Research & Center of Neurology, University of Tuebingen, Tuebingen, Germany (site investigator). Georgios Koutsis: Department of Neurology, Eginition Hospital, National and Kapodistrian University of Athens, Athens, Greece (principal investigator). Richard Walsh: NationalAtaxia Clinic, Department of Neurology, Adelaide & Meath Hospital Dublin, incorporating the National Children's Hospital, Dublin, Ireland (principal investigator). Enrico Bertini: Department of Neurosciences, Unit of Neuromuscular and Neurodegenerative Disorders, Bambino, Gesu’ Children's Research Hospital, IRCCS, Rome, Italy (principal investigator).
