# Supplementary figures and images for: Increase of Plasma Biomarkers in Friedreich's Ataxia: Potential Insights into Disease Pathology
Source: Mov Disord. 2025 Jun 11;40(9):1863–73. doi: 10.1002/mds.30250 (PMC12485593; doi:10.1002/mds.30250)

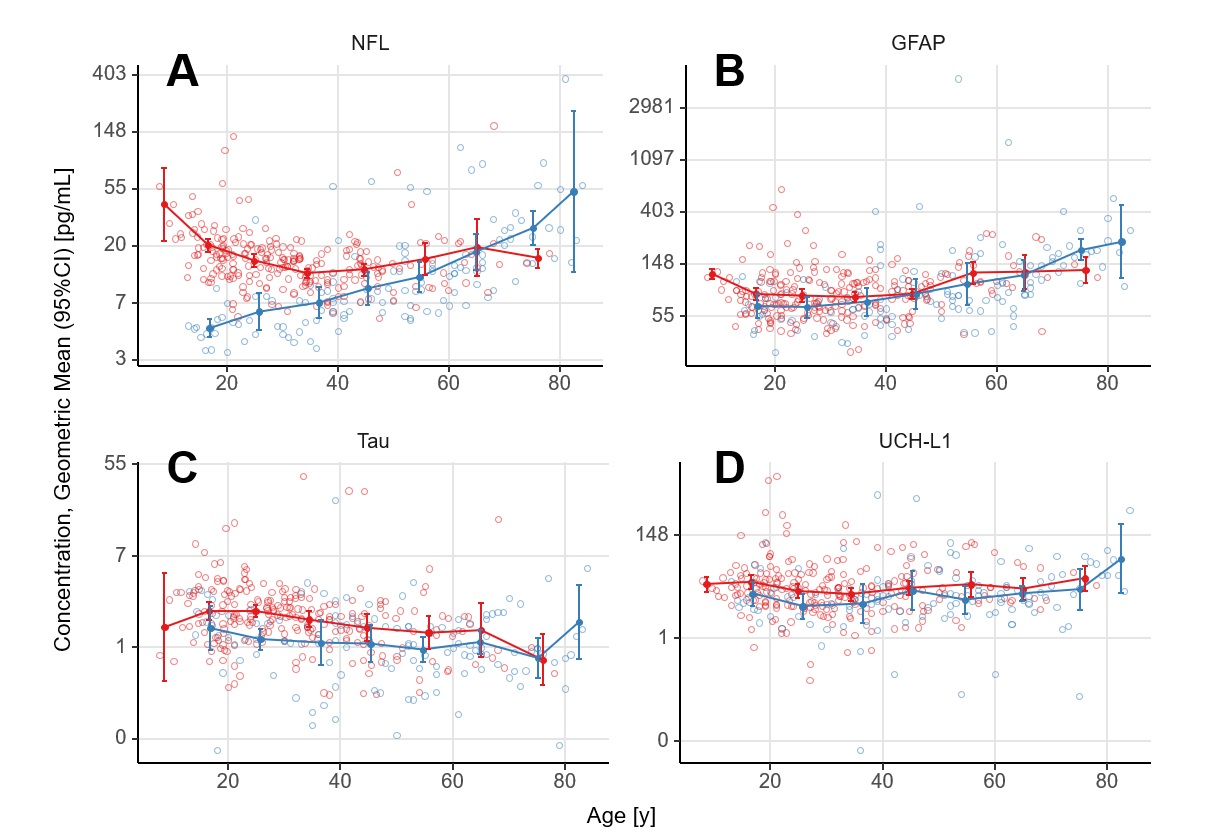

Supplement: Supplementary file 1 — FIGURE S1. Absolute biomarker levels in participants with FRDA (red) and controls (blue) by age. Mean lines for samples within 5 years‐age bins are depicted in connected dots with 95% CIs; individual measures are shown as circles (A: NfL, B: FAP, C: t‐tau and D: UCH‐L1). [file MDS-40-1863-s002.jpg]

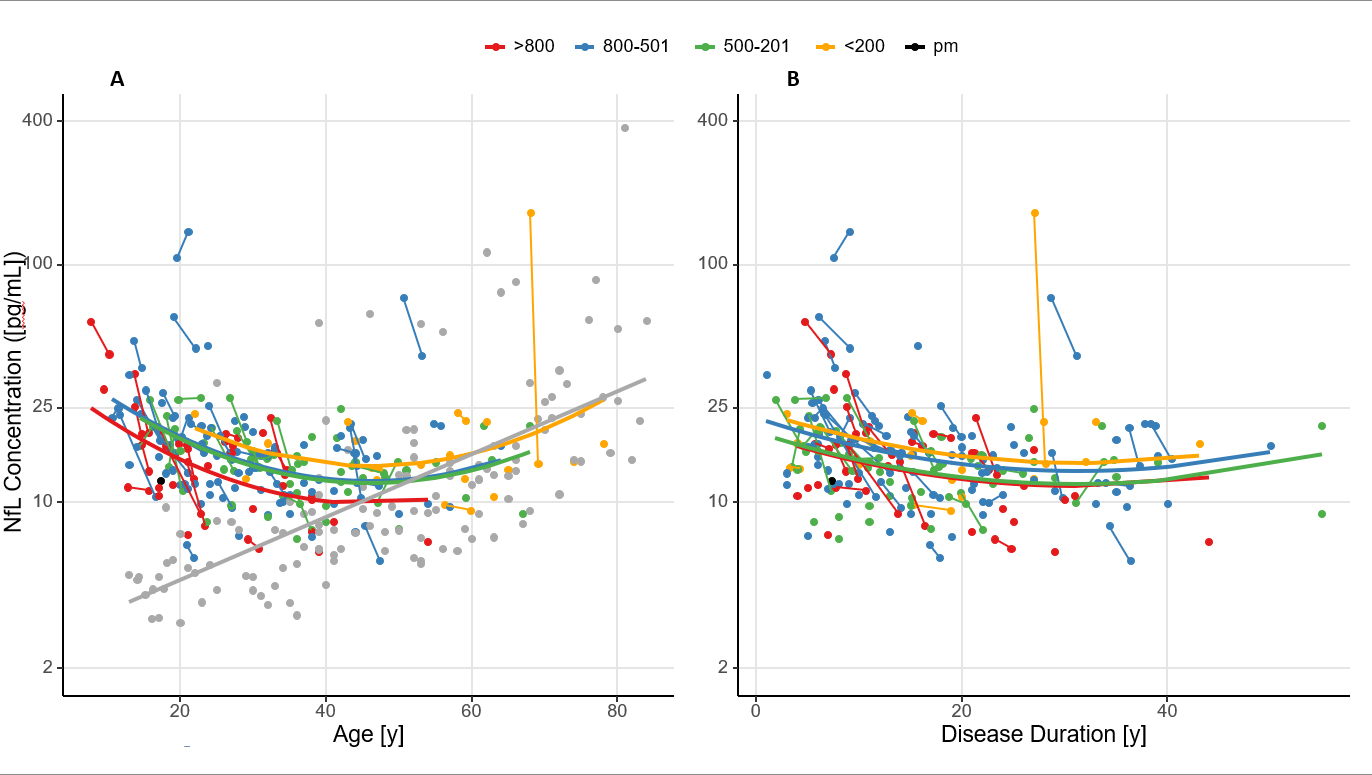

Supplement: Supplementary file 2 — FIGURE S2. Absolute NfL Levels in controls (grey) and FRDA patients relative to age (A) and disease duration (B). FRDA patients were grouped and coloured by GAA1 repeat length. Solid lines depict polynomial model functions of NfL levels, by respective GAA1‐repeat length group. Control levels were modelled using a linear function over age. [file MDS-40-1863-s001.jpg]
