# Supplementary material for: Increase of Plasma Biomarkers in Friedreich's Ataxia: Potential Insights into Disease Pathology
Source: Mov Disord. 2025 Jun 11;40(9):1863–73. doi: 10.1002/mds.30250 (PMC12485593; doi:10.1002/mds.30250)
Supplement: Supplementary file 3 — Table S1. Yearly Rates of change and age adjusted differences in NfL, GFAP, t‐tau and UCH‐L1 using linear mixed effects modelling. [file MDS-40-1863-s003.docx]

| Biomarker | Group | Rate of Change by age  (log pg/mL) (95%CI) | p | Age adjusted difference  FRDA vs controls (log pg/mL)  (95%CI) | p |
| --- | --- | --- | --- | --- | --- |
| NfL | FRDA | -0.007  (-0.012, -0.003) | 0.001 | -0.497 (-0.639, -0.355) | < 0.001 |
|  | control | 0.031 (0.025, 0.036) | < 0.001 |  |  |
| GFAP | FRDA | 0.007  (0.002, 0.011) | 0.002 | -0.096 (-0.229, 0.038) | 0.163 |
|  | control | 0.018 (0.012, 0.024) | < 0.001 |  |  |
| t-tau | FRDA | -0.014  (-0.020, -0.007) | < 0.001 | -0.384 (-0.561, -0.206) | < 0.001 |
|  | control | -0.006 (-0.013, 0.002) | 0.127 |  |  |
| UCH-L1 | FRDA | -0.005 (-0.017, 0.007) | 0.396 | -0.359 (-0.706, -0.011) | 0.044 |
|  | control | 0.013  (-0.002, 0.028) | 0.087 |  |  |

Supplementary Table 1
