# Supplementary material for: Increase of Plasma Biomarkers in Friedreich's Ataxia: Potential Insights into Disease Pathology
Source: Mov Disord. 2025 Jun 11;40(9):1863–73. doi: 10.1002/mds.30250 (PMC12485593; doi:10.1002/mds.30250)
Supplement: Supplementary file 4 — Table S2. Annualized change in geometric mean ratio of NfL using follow‐up samples, analyzed by pediatric, young adult, and older adult age groups. [file MDS-40-1863-s004.docx]

Supplementary Table 2

| **Age Group** | **N Patients** | **Mean (SD) NfL Change (pg/mL/year)** | **GMR per year** | **Interpretation** |
| --- | --- | --- | --- | --- |
| Children, <18y | 15 | –3.5 (3.6) | 0.87 | 13% annual decrease |
| Adults 18-35y | 33 | –1.3 (2.5) | 0.93 | 7% annual decrease |
| Adults > 35y | 13 | -1.5 (2.3) | 0.90 | 10% annual decrease |
