# Supplementary material for: Increase of Plasma Biomarkers in Friedreich's Ataxia: Potential Insights into Disease Pathology
Source: Mov Disord. 2025 Jun 11;40(9):1863–73. doi: 10.1002/mds.30250 (PMC12485593; doi:10.1002/mds.30250)
Supplement: Supplementary file 5 — Data S1. Supporting Information. [file MDS-40-1863-s005.docx]

SCA 3

- Wilke C, Haas E, Reetz K, et al. Neurofilaments in spinocerebellar ataxia type 3: blood biomarkers at the preataxic and ataxic stage in humans and mice. EMBO Mol. Med. 2020;12(7):e11803.


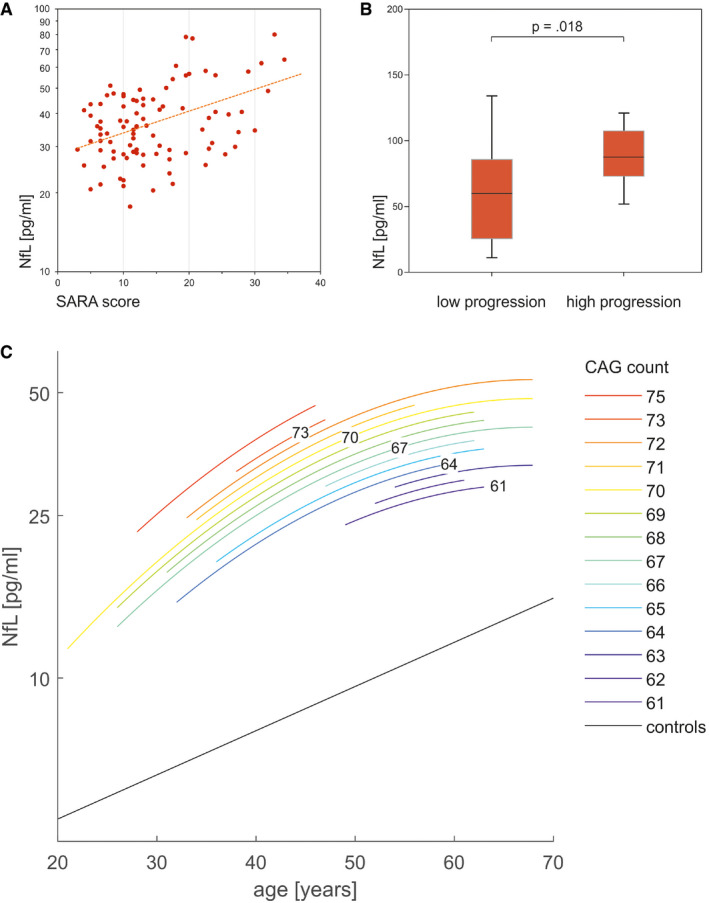


- Garcia-Moreno H, Prudencio M, Thomas-Black G, et al. Tau and neurofilament light-chain as fluid biomarkers in spinocerebellar ataxia type 3. Eur. J. Neurol. 2022;29(8):2439–2452.


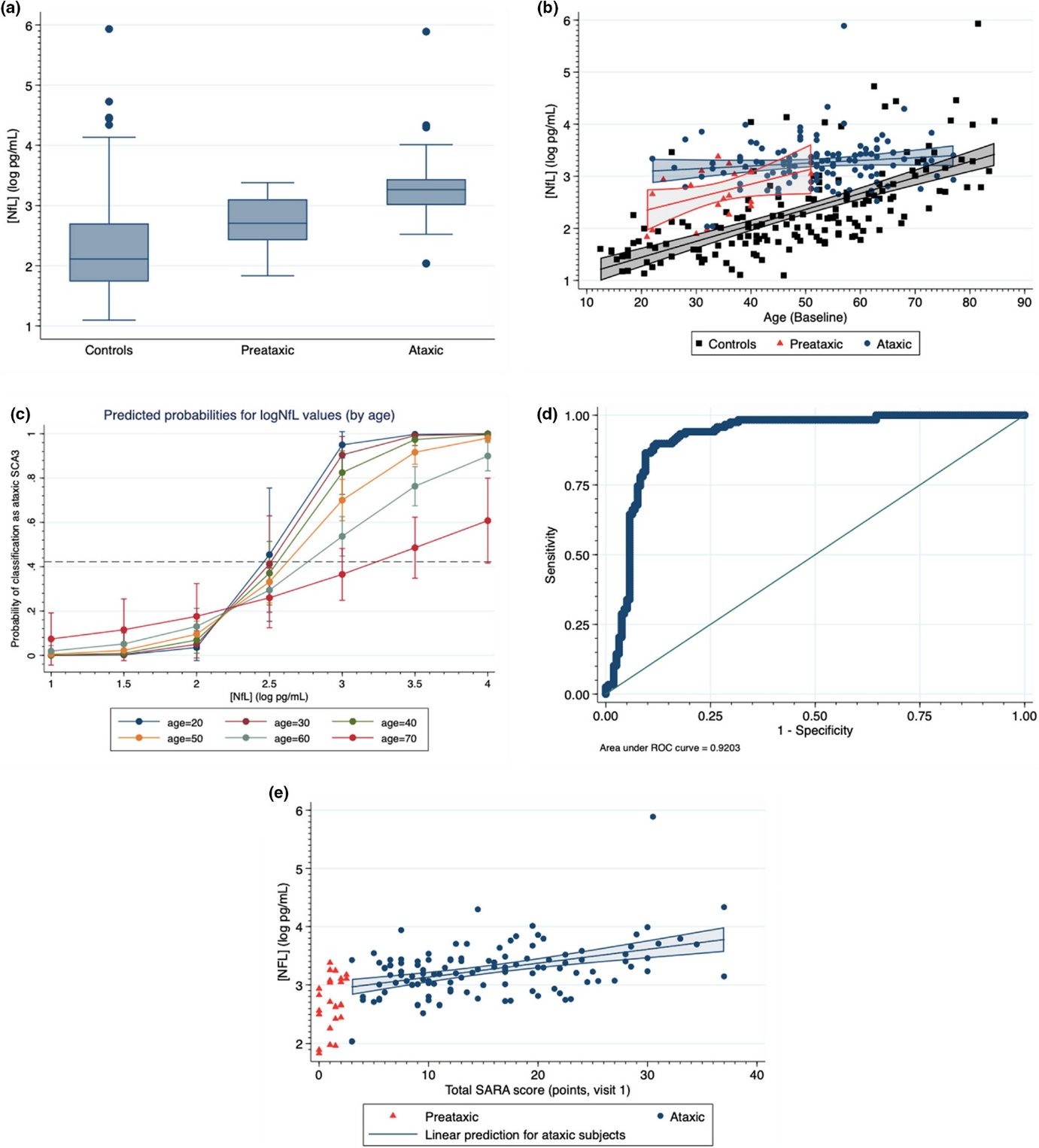


A-T

Donath H, Woelke S, Schubert R, et al. Neurofilament Light Chain Is a Biomarker of Neurodegeneration in Ataxia Telangiectasia [published correction appears in Cerebellum. 2022 Feb;21(1):48. doi: 10.1007/s12311-021-01280-5]. *Cerebellum*. 2022;21(1):39-47. doi:10.1007/s12311-021-01257-4


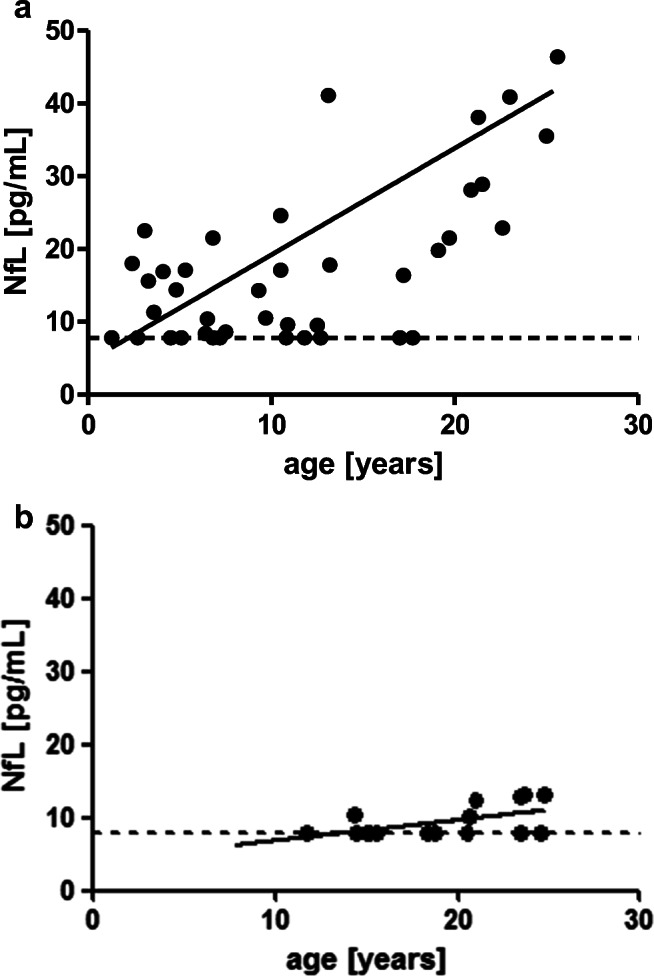


CJD

Schmitz M, Canaslan S, Espinosa JC, et al. Validation of Plasma and CSF Neurofilament Light Chain as an Early Marker for Sporadic Creutzfeldt-Jakob Disease. *Mol Neurobiol*. 2022;59(9):1-9. doi:10.1007/s12035-022-02891-7


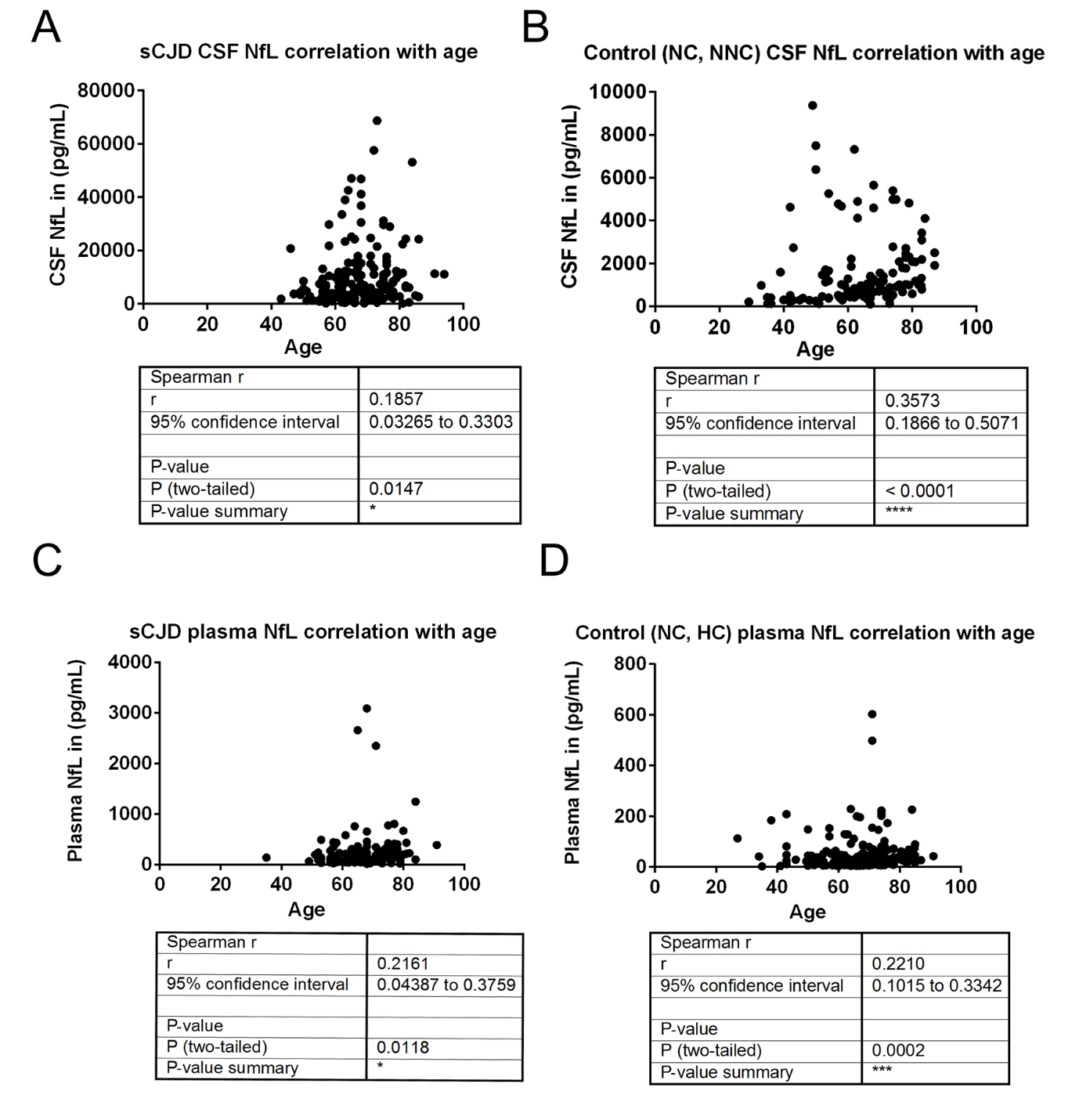


CMT

Sandelius Å, Zetterberg H, Blennow K, et al. Plasma neurofilament light chain concentration in the inherited peripheral neuropathies. *Neurology*. 2018;90(6):e518-e524. doi:10.1212/WNL.0000000000004932


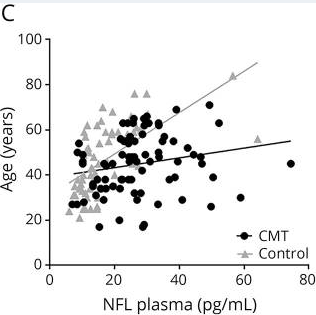


Huntington’s Disease

Byrne LM, Rodrigues FB, Blennow K, et al. Neurofilament light protein in blood as a potential biomarker of neurodegeneration in Huntington's disease: a retrospective cohort analysis [published correction appears in Lancet Neurol. 2017 Sep;16(9):683. doi: 10.1016/S1474-4422(17)30255-7]. *Lancet Neurol*. 2017;16(8):601-609. doi:10.1016/S1474-4422(17)30124-2


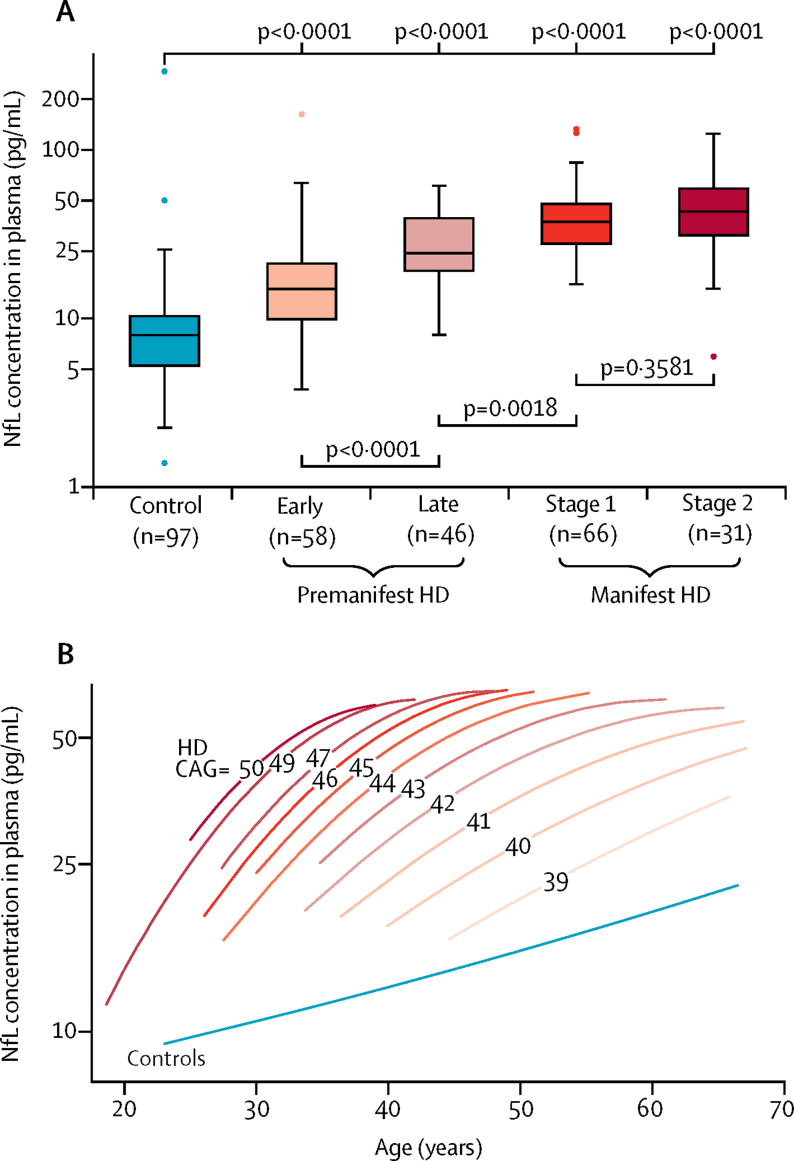


RFC1 Ataxia

Quartesan I, Vegezzi E, Currò R, et al. Serum Neurofilament Light Chain in Replication Factor Complex Subunit 1 CANVAS and Disease Spectrum. *Mov Disord*. 2024;39(1):209-214. doi:10.1002/mds.29680


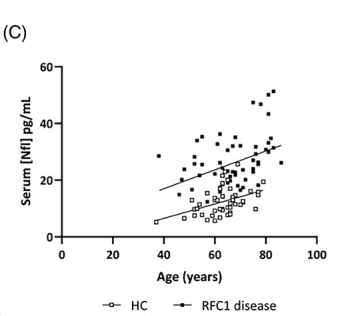


Autosomal Dominant Alzheimer’s Disease

Hofmann A, Häsler LM, Lambert M, et al. Comparative neurofilament light chain trajectories in CSF and plasma in autosomal dominant Alzheimer's disease. *Nat Commun*. 2024;15(1):9982. Published 2024 Nov 18. doi:10.1038/s41467-024-52937-8


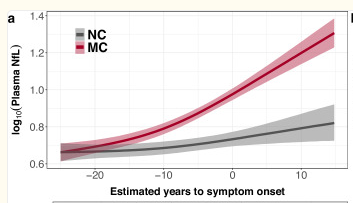


MSA

Oender D, Faber J, Wilke C, et al. Evolution of Clinical Outcome Measures and Biomarkers in Sporadic Adult-Onset Degenerative Ataxia. *Mov Disord*. 2023;38(4):654-664. doi:10.1002/mds.29324


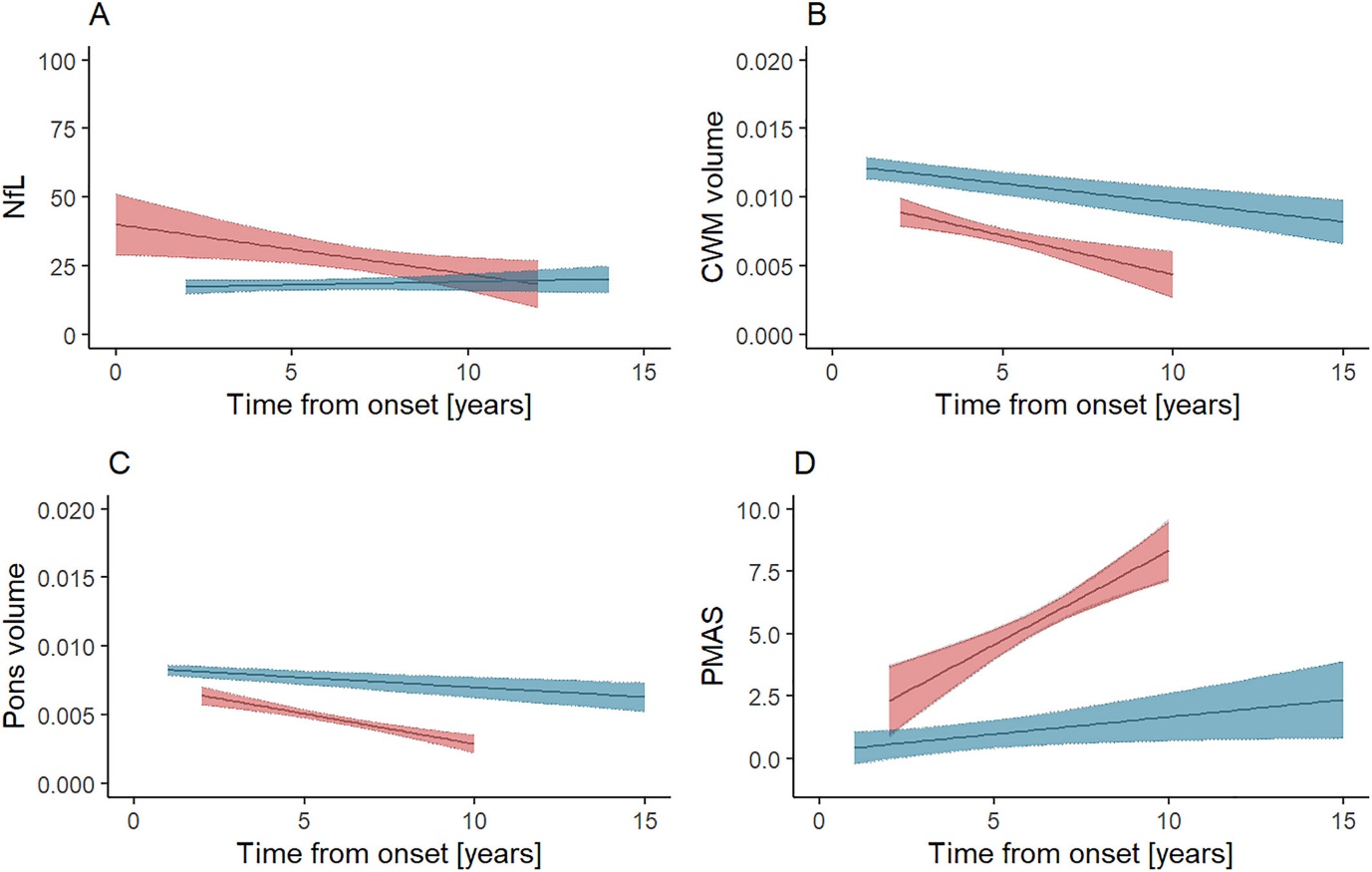


Chelban V, Nikram E, Perez-Soriano A, et al. Neurofilament light levels predict clinical progression and death in multiple system atrophy. *Brain*. 2022;145(12):4398-4408. doi:10.1093/brain/awac253


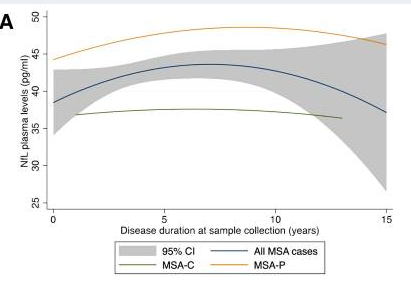


Niemann Pick Type C

Dardis A, Pavan E, Fabris M, et al. Plasma Neurofilament Light (NfL) in Patients Affected by Niemann-Pick Type C Disease (NPCD). *J Clin Med*. 2021;10(20):4796. Published 2021 Oct 19. doi:10.3390/jcm10204796


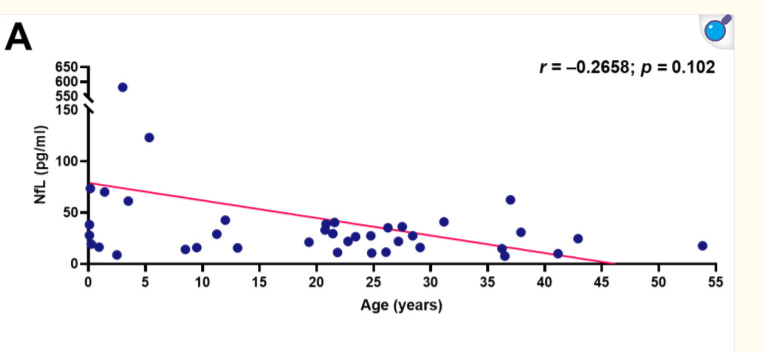


Down Syndrome

NF-L level and age of participants were significantly correlated (Spearman’s rho = 0.789, p < 0.001) (Fig. 1), such that those aged 35 and older had significantly higher levels of NF-L than younger individuals (median 11.52 ng/L vs. 32.42 ng/L, Mann-Whitney U test p < 0.001).

Strydom, A., Heslegrave, A., Startin, C.M. *et al.* Neurofilament light as a blood biomarker for neurodegeneration in Down syndrome. *Alz Res Therapy* **10**, 39 (2018). <https://doi.org/10.1186/s13195-018-0367-x>


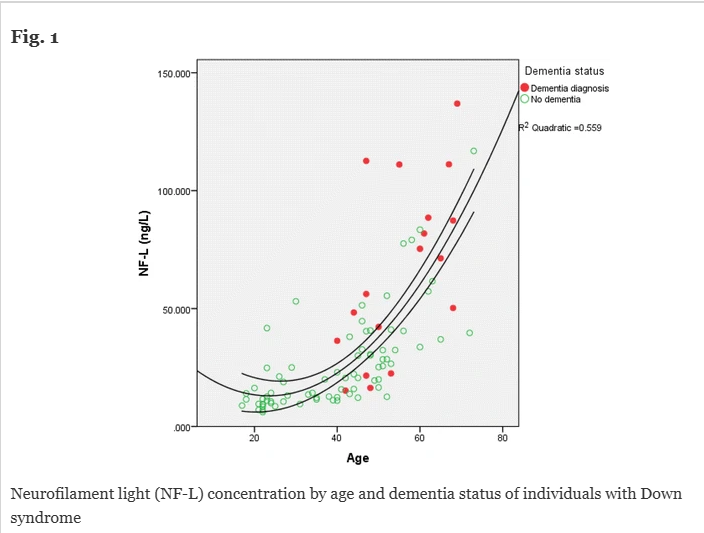


ALS

Benatar M, Wuu J, Andersen PM, Lombardi V, Malaspina A. Neurofilament light: A candidate biomarker of presymptomatic amyotrophic lateral sclerosis and phenoconversion. *Ann Neurol*. 2018;84(1):130-139. doi:10.1002/ana.25276

Longitudinally, serum NfL levels were essentially stable in controls and ALS patients = although there are as yet insufficient data to reliably estimate the slopes in a mixed-model analysis. By contrast, in at-risk individuals, adjusting for baseline age, serum NfL increased by an average of 2.41pg/ml per 10-year increase in age (p = 0.004). Moreover, among converters, elevated NfL levels (ie, above the highest value observed in controls) were observed as far back as 11.6 months preceding phenoconversion, and their NfL levels continued to increase through at least the first 6 months after symptom onset.


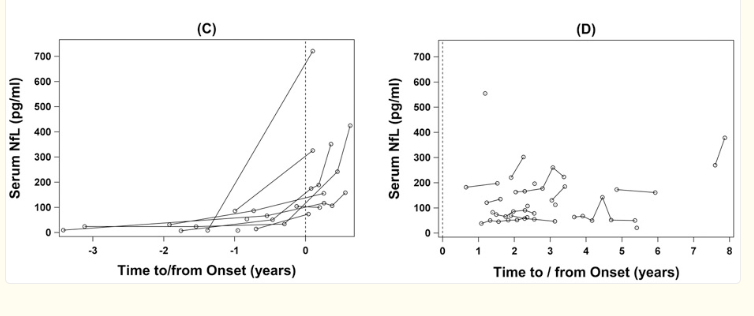


C- phenoconverters

D- ALS patients

SMA


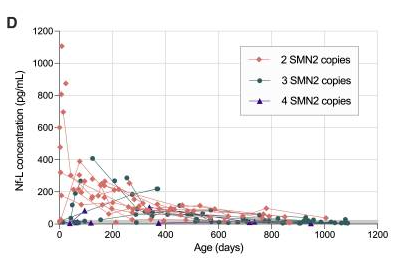


Fewer copies often more severe in SMA

Alves CRR, Petrillo M, Spellman R, et al. Implications of circulating neurofilaments for spinal muscular atrophy treatment early in life: A case series. *Mol Ther Methods Clin Dev*. 2021;23:524-538. Published 2021 Oct 30. doi:10.1016/j.omtm.2021.10.011

Wilson’s Disease

There were no significant correlations between age, courses of disease and plasma NfL concentrations in the WD group (p > 0.05)

Shribman, S., Heller, C., Burrows, M., Heslegrave, A., Swift, I., Foiani, M.S., Gillett, G.T., Tsochatzis, E.A., Rowe, J.B., Gerhard, A., Butler, C.R., Masellis, M., Bremner, F., Martin, A., Jung, L., Cook, P., Zetterberg, H., Bandmann, O., Rohrer, J.D. and Warner, T.T. (2021), Plasma Neurofilament Light as a Biomarker of Neurological Involvement in Wilson's Disease. Mov Disord, 36: 503-508.

| Disease(s) | Relationship | Reference(s) |
| --- | --- | --- |
| SCA 3 | Statistically significant increase with increasing age, higher than controls at all ages | - Wilke C, Haas E, Reetz K, et al. Neurofilaments in spinocerebellar ataxia type 3: blood biomarkers at the preataxic and ataxic stage in humans and mice. EMBO Mol. Med. 2020;12(7):e11803.  - Garcia-Moreno H, Prudencio M, Thomas-Black G, et al. Tau and neurofilament light-chain as fluid biomarkers in spinocerebellar ataxia type 3. Eur. J. Neurol. 2022;29(8):2439–2452. |
| AT | There was a significant correlation of NfL with age in A-T patients (r = 0.45, p ≤ 0.01) | Donath H, Woelke S, Schubert R, et al. Neurofilament Light Chain Is a Biomarker of Neurodegeneration in Ataxia Telangiectasia [published correction appears in Cerebellum. 2022 Feb;21(1):48. doi: 10.1007/s12311-021-01280-5]. *Cerebellum*. 2022;21(1):39-47. doi:10.1007/s12311-021-01257-4 |
| CJD | The correlation analysis indicated a significant correlation between NfL with age in the sCJD and the control groups | Schmitz M, Canaslan S, Espinosa JC, et al. Validation of Plasma and CSF Neurofilament Light Chain as an Early Marker for Sporadic Creutzfeldt-Jakob Disease. *Mol Neurobiol*. 2022;59(9):1-9. doi:10.1007/s12035-022-02891-7 |
| CMT | Plasma NfL concentration correlated with age and this was more pronounced in controls than in patients with CMT (control: r = 0.70, p < 0.0001; CMT: r = 0.28, p = 0.012; Fisher r to z transformation (p < 0.0001) | Sandelius Å, Zetterberg H, Blennow K, et al. Plasma neurofilament light chain concentration in the inherited peripheral neuropathies. *Neurology*. 2018;90(6):e518-e524. doi:10.1212/WNL.0000000000004932 |
| HD | We found positive associations between NfL concentrations in plasma and age in controls and all Huntington's disease subgroups. In controls, the association was roughly linear (slope 0·02 log pg/mL per year [SE 0·0042], p<0·0001 | Byrne LM, Rodrigues FB, Blennow K, et al. Neurofilament light protein in blood as a potential biomarker of neurodegeneration in Huntington's disease: a retrospective cohort analysis [published correction appears in Lancet Neurol. 2017 Sep;16(9):683. doi: 10.1016/S1474-4422(17)30255-7]. *Lancet Neurol*. 2017;16(8):601-609. doi:10.1016/S1474-4422(17)30124-2 |
| RFC1 | Serum NfL concentration showed a moderate correlation with age in both HCs (r = 0.4353, P = 0.0020) and patients (r = 0.4092, P = 0.0011) | Quartesan I, Vegezzi E, Currò R, et al. Serum Neurofilament Light Chain in Replication Factor Complex Subunit 1 CANVAS and Disease Spectrum. *Mov Disord*. 2024;39(1):209-214. doi:10.1002/mds.29680 |
| Autosomal Dominant AD | Cross-sectionally, NfL levels in CSF and blood in the MC group began to increase, compared to NC group, between 15-25 years prior to expected symptom onset | Hofmann A, Häsler LM, Lambert M, et al. Comparative neurofilament light chain trajectories in CSF and plasma in autosomal dominant Alzheimer's disease. *Nat Commun*. 2024;15(1):9982. Published 2024 Nov 18. doi:10.1038/s41467-024-52937-8 |
| MSA | Mixed-effects modeling revealed a mild decrease of NfL levels in MSA-C **with disease duration**  Plasma NfL was positively associated with age at sample collection in MSA patients (rho = 0.21, P = 0.01)  In the early disease stage, an increase in disease duration was associated with an increase in NfL concentration in plasma followed by a short plateau. At ∼7 years of **disease duration**, a decrease in NfL levels is observed. | Oender D, Faber J, Wilke C, et al. Evolution of Clinical Outcome Measures and Biomarkers in Sporadic Adult-Onset Degenerative Ataxia. *Mov Disord*. 2023;38(4):654-664. doi:10.1002/mds.29324  Chelban V, Nikram E, Perez-Soriano A, et al. Neurofilament light levels predict clinical progression and death in multiple system atrophy. *Brain*. 2022;145(12):4398-4408. doi:10.1093/brain/awac253 |
| NPC | In contrast to healthy controls, no correlation between plasma NfL levels and age at sampling was found in NPCD patients (Pearson’s correlation coefficient = −0.2658; p = 0.102) | Dardis A, Pavan E, Fabris M, et al. Plasma Neurofilament Light (NfL) in Patients Affected by Niemann-Pick Type C Disease (NPCD). *J Clin Med*. 2021;10(20):4796. Published 2021 Oct 19. doi:10.3390/jcm10204796 |
| Down Syndrome | those aged 35 and older had significantly higher levels of NF-L than younger individuals (median 11.52 ng/L vs. 32.42 ng/L, Mann-Whitney *U* test *p* < 0.001). | Strydom, A., Heslegrave, A., Startin, C.M. *et al.* Neurofilament light as a blood biomarker for neurodegeneration in Down syndrome. *Alz Res Therapy* **10**, 39 (2018). https://doi.org/10.1186/s13195-018-0367-x |
| Wilson’s Disease | There were no significant correlations between age, courses of disease and plasma NfL concentrations in the WD group (p > 0.05) | Shribman, S., Heller, C., Burrows, M., Heslegrave, A., Swift, I., Foiani, M.S., Gillett, G.T., Tsochatzis, E.A., Rowe, J.B., Gerhard, A., Butler, C.R., Masellis, M., Bremner, F., Martin, A., Jung, L., Cook, P., Zetterberg, H., Bandmann, O., Rohrer, J.D. and Warner, T.T. (2021), Plasma Neurofilament Light as a Biomarker of Neurological Involvement in Wilson's Disease. Mov Disord, 36: 503-508. |
| SMA | The highest Nf-L value observed in a healthy control subject was ∼20 pg/mL, while up to 1,100 pg/mL was observed in SMA patients. SMA patients with 2 *SMN2* copies also had higher Nf-L levels than healthy controls and those SMA patients with 4 *SMN2* copies or 3 *SMN2* copies. SMA patients with 3 *SMN2* copies also had higher Nf-L levels than healthy controls, and those SMA patients with 4 *SMN2* copies.  *No analysis/comment made on evolution of NfL with age in this cohort* | Alves CRR, Petrillo M, Spellman R, et al. Implications of circulating neurofilaments for spinal muscular atrophy treatment early in life: A case series. *Mol Ther Methods Clin Dev*. 2021;23:524-538. Published 2021 Oct 30. doi:10.1016/j.omtm.2021.10.011 |
| ALS | Longitudinally, serum NfL levels were essentially stable in controls and ALS patients, although there are as yet insufficient data to reliably estimate the slopes in a mixed-model analysis. By contrast, in at-risk individuals, adjusting for baseline age, serum NfL increased by an average of 2.41pg/ml per 10-year increase in age (p = 0.004). Moreover, among converters, elevated NfL levels (ie, above the highest value observed in controls) were observed as far back as 11.6 months preceding phenoconversion, and their NfL levels continued to increase through at least the first 6 months after symptom onset. | Benatar M, Wuu J, Andersen PM, Lombardi V, Malaspina A. Neurofilament light: A candidate biomarker of presymptomatic amyotrophic lateral sclerosis and phenoconversion. *Ann Neurol*. 2018;84(1):130-139. doi:10.1002/ana.25276 |
